# Electroacupuncture Pretreatment Ameliorates PTSD-Like Behaviors in Rats by Enhancing Hippocampal Neurogenesis via the Keap1/Nrf2 Antioxidant Signaling Pathway

**DOI:** 10.3389/fncel.2019.00275

**Published:** 2019-06-21

**Authors:** Cui-hong Zhou, Fen Xue, Shan-shan Xue, Han-fei Sang, Ling Liu, Ying Wang, Min Cai, Zhang-Jin Zhang, Qing-rong Tan, Hua-ning Wang, Zheng-wu Peng

**Affiliations:** ^1^Xijing Hospital, Fourth Military Medical University, Xi’an, China; ^2^Department of Anesthesiology, Xiang’an Hospital, Xiamen, China; ^3^Institution of Neuroscience, Fourth Military Medical University, Xi’an, China; ^4^School of Chinese Medicine, LKS Faculty of Medicine, The University of Hong Kong, Hong Kong, China

**Keywords:** electroacupuncture, pretreatment, post-traumatic stress disorder, hippocampus, keap1/Nrf2

## Abstract

Electroacupuncture (EA) pretreatment is a clinically useful therapy for several brain disorders. However, whether and via which exact molecular mechanisms it ameliorates post-traumatic stress disorder (PTSD) remains unclear. In the present study, rats received EA stimulation for seven consecutive days before exposure to enhanced single prolonged stress (ESPS). Anxiety-like and fear learning behaviors; hippocampal neurogenesis; the expression of nuclear factor erythroid 2-related factor 2 (Nrf2), Kelch-like ECH-associated protein 1 (keap1), and heme oxygenase 1 (HO-1); and the activity of AMP-activated kinase (AMPK) were evaluated at 14 days after ESPS. EA pretreatment improved hippocampal neurogenesis and ameliorated anxiety-like behaviors in ESPS-treated rats. EA pretreatment also increased the expression of Nrf2 and HO-1 and the activity of AMPK. Furthermore, Nrf2 knockdown by a short hairpin RNA affected anxiety-like behaviors and expression of neuroprotective markers (BDNF, DCX) in a manner similar to ESPS alone and dampened the neuroprotective effects of EA pretreatment. In contrast, Keap1 knockdown increased the expression of HO-1, improved hippocampal neurogenesis, and alleviated PTSD-like behaviors. Altogether, our results suggest that EA pretreatment ameliorates ESPS-induced anxiety-like behaviors and prevents hippocampal neurogenesis disruption in a rat model of PTSD possibly through regulation of the keap1/Nrf2 antioxidant defense pathway.

## Introduction

Post-traumatic stress disorder (PTSD) is a fear-based biopsychosocial disorder that is caused by exposure to severely traumatic events, such as sexual violence, war, and life-threatening accidents (Careaga et al., 2016). PTSD severely affects patients’ quality of life and social stability. Epidemiological studies have revealed that more than half of the world’s population experiences stressful events and that the lifetime and average prevalence of PTSD are 6.8% and 8%, respectively (McLaughlin et al., 2015; Liu et al., 2017). Currently, PTSD is mainly treated with psychological intervention and drugs. However, none of these approaches have proved to constitute a satisfying method to improve the clinical symptoms of these patients (Sripada et al., 2016). Thus, the development of novel treatment strategies is an urgent need in PTSD.

It is well known that the hippocampus plays a crucial role in memory processes and fear conditioning responses—highly relevant phenomena to the pathogenesis of PTSD (Corcoran and Maren, 2001; Kheirbek et al., 2013; Girardeau et al., 2017). Physical or psychosocial stressors may induce morphological changes in the hippocampus, including reduced neurogenesis and loss of pyramidal neurons (Cohen et al., 2014). Patients with PTSD have reduced hippocampal volumes (Filipovic et al., 2011) and animal models of PTSD show suppressed hippocampal cell proliferation, inhibited neurogenesis, and increased neuronal apoptosis (Peng et al., 2013). It has been suggested that brain oxidative damage may be the cause of hippocampal structure and function impairments (Miller and Sadeh, 2014). In the hippocampus of PTSD-like rats, total reactive oxygen species (ROS), peroxynitrite and superoxide levels are elevated (Wilson et al., 2013). Moreover, cranially irradiated superoxide dismutase (SOD)-deficient mice exhibit decreased neurogenesis in the hippocampus due to long-term ROS accumulation (Yuan et al., 2015). Interestingly, preclinical studies have also shown that antioxidants can improve hippocampal neurogenesis and attenuate anxiety-like behaviors in animal models (Bouayed et al., 2009; Moustafa, 2013). One may then hypothesize that inhibiting oxidative damage may improve hippocampal function and be beneficial for treating PTSD.

Nuclear factor erythroid 2-related factor 2 (Nrf2) and its antioxidant signaling pathway are key regulators of neuroprotection against oxidative stress (Mitsuishi et al., 2012; Ahuja et al., 2016). The activation of Nrf2 confers protective effects to many central nervous system diseases (Gan and Johnson, 2014), and Nrf2 silencing in the brain increases anxiety-like behaviors in rats (Khalifeh et al., 2015). Under physiological conditions, Nrf2 binds to Kelch-like ECH-associated protein 1 (keap1) in the cytoplasm. However, upon exposure to ROS, Nrf2 disassociates from keap1 and then translocates to the nucleus, where it activates the transcription of several antioxidant enzymes genes, including *SOD* and heme oxygenase 1 (*HO-1*) (Kubben et al., 2016; Cai et al., 2017), which protect hippocampal neurons against oxidative stress (Lee et al., 2015). A recent study has shown that Nrf2 activation in lipopolysaccharide-treated mice or cells is accompanied by an increase in the phosphorylation of AMP-activated kinase (AMPK) and inhibition of AMPK blocked aucubin-induced expression of Nrf2 and its downstream effector HO-1 (Qiu et al., 2018). Taken together, inhibition of oxidative damage via activation of the keap1/Nrf2 antioxidant defense pathway may improve hippocampal function and be beneficial for treating PTSD.

Electroacupuncture (EA) combines the advantages of acupuncture and electrophysiological stimulation. Its beneficial effects, including during the pretreatment phase, have been demonstrated for several neuropsychiatric disorders. Although the precise mechanisms remain to be fully elucidated, enhanced neurogenesis and synaptic plasticity, and prevention of oxidative damage and inflammation have been described following EA pretreatment in previous studies (Feng et al., 2010; Chen et al., 2012, 2016). It has been suggested that the keap1/Nrf2 pathway may be involved in the protective effect of EA (Yu et al., 2014, 2015). Activation of both AMPK and HO-1 is required for EA to exert its therapeutic effects (Yu et al., 2014, 2015). Thus, we hypothesize that EA pretreatment is beneficial for the prevention of PTSD and that the keap1/Nrf2 pathway might play a role in this process. In this study, we sought to determine whether EA pretreatment could ameliorate stress-associated behaviors in a rat model of PTSD. We also aimed to examine whether changes in the activity of keap1/Nrf2 and its downstream antioxidative proteins in the hippocampus could be involved in the EA pretreatment effects.

## Materials and Methods

### Animals

Male Sprague–Dawley rats (280–320 g) were obtained from the Animal Center of Fourth Military Medical University (FMMU). Rats were group-housed (four per cage) and maintained at 20–25°C on a 12-h light/dark daily cycle with free access to food and water. The experiment procedures were in accordance with the National Institutes of Health Guide for the Care and Use of Laboratory Animals and were approved by the Animal Use and Protection Committee of the FMMU.

### Experimental Design

#### Experiment I

To determine the effect of EA pretreatment on PTSD, after 7 days of acclimatization, 32 rats were randomly divided into four groups (eight rats per group): control, EA, enhanced single prolonged stress (ESPS), and EA + ESPS. Rats in the control group were given false stimulation (EA treatment without electricity) for seven consecutive days (30 min every day) and then housed in their home cage for 2 weeks. Rats in the EA and EA + ESPS groups were stimulated with EA at a frequency of 2/15 Hz and an intensity of 1 mA for seven consecutive days (30 min every day). Rats in the ESPS group were given false stimulation (EA treatment without electricity) for seven consecutive days and then subjected to ESPS. The researchers performing the behavioral testing were blinded to the animals’ group allocation. Then, hippocampal neurogenesis, the expression of BDNF, DCX, Nrf2, and HO-1, as well as the activity of AMPK were evaluated.

#### Experiment II

To investigate the role of the Nrf2 antioxidant signaling pathway in the hippocampus in the neuroprotective effect of EA pretreatment, 64 rats were randomly divided into eight groups (eight rats per group): Scramble, Scramble + ESPS, Scramble + EA + ESPS, shNrf2, shNrf2 + ESPS, shNrf2 + EA, shNrf2 + EA + ESPS, and shkeap1 + ESPS. Rats in the Scramble, Scramble + ESPS, and Scramble + EA + ESPS groups were injected with scramble short hairpin RNA (shRNA) (a lentivirus carrying scramble shRNA), while rats in the shNrf2, shNrf2 + ESPS, shNrf2 + EA, shNrf2 + EA + ESPS, and shkeap1 + ESPS groups were injected with Nrf2-shRNA or keap1-shRNA lentivirus in the hippocampus. Two weeks after the lentivirus injection, rats in the Scramble + EA + ESPS, shNrf2 + EA and shNrf2 + EA + ESPS groups received EA stimulation (2/15 Hz, 1 mA) for seven consecutive days (30 min every day), while rats in the Scramble, Scramble + ESPS, shNrf2, shNrf2 + ESPS and shkeap1 + ESPS groups received false stimulation as described in Experiment I, after which rats in the Scramble + ESPS, Scramble + EA + ESPS, shNrf2 + ESPS, shNrf2 + EA + ESPS, and shkeap1 + ESPS groups were subjected to ESPS. The behavioral tests were performed 2 weeks after ESPS. Then, hippocampal neurogenesis and gene expression were determined as in Experiment I.

### EA Treatment

Electroacupuncture treatment was performed as described previously (Feng et al., 2010; Wang et al., 2014). Briefly, rats were maintained on a platform (10 cm × 10 cm × 50 cm) without anesthesia and the acupoint “Bai hui” (GV20), which is located at the intersection of the sagittal midline and the line linking the rat ears, was stimulated for 30 min daily (frequency: 2/15 Hz, waveform: dilatational wave, intensity: 1 mA) by using the G6805–2 EA instrument (No. 227033; Qingdao Xinsheng Ltd.). False stimulation was performed at the same acupoint without electricity.

### Enhanced Single Prolonged Stress (ESPS)

Enhanced single prolonged stress was performed in accordance with our previous study (Wang H. N. et al., 2015). Rats were restrained for 2 h and then immediately exposed to forced swimming in water (diameter: 24 cm, height: 50 cm, water temperature: 24°C) for 20 min and then exposed to diethyl ether until they lost consciousness after recuperation for 15 min. Finally, rats were exposed to a single electric foot shock (1 mA for 4 s) after 30 min of recovering in a rectangular box with stainless steel rods floors and aluminum and acrylic walls.

### Behavioral Tests

All the behavioral tests began 14 days after ESPS exposure. Rats were acclimatized to the separate experimental room for at least 30 min prior to each test, and all experiments were conducted under low light conditions in order to minimize anxiety effects. The area was cleaned with 75% ethanol between tests. Besides, the open field test was conducted prior to the elevated plus maze test on the same day, while the fear conditioning test was performed 24 h after the elevated plus maze test.

### Open Field Test (OFT)

According to previous studies (Sullivan et al., 2003; Missault et al., 2019), the OFT was used to assess anxiety-related behavior and locomotor activity in an open field arena (47 cm × 47 cm × 50 cm). Rats were gently placed in one of the arena’s corners and recorded from the soundproof box, which was illuminated by a red fluorescent light (30 W). After each trial, the apparatus was cleansed with 75% ethanol. The time spent in the center of the arena that could be used for the quantification of rodent anxiety and exploratory drive was recorded for 10 min and analyzed by using an automatic system (Top Scan, Clever Sys Inc., United States). Rats with high levels of innate anxiety typically avoid the center arena and spend more time in close proximity to the walls. The total distance moved in the open field was also measured to analyze general locomotor activity according to previous work (Yang et al., 2016).

### Elevated Plus Maze Test (EPMT)

The EPMT has been well validated for detecting anxiety-like behavior. The Plexiglas apparatus (Dig Behav, Ji Liang Co., Ltd., Shanghai, China) consisted of two opposite open arms (50 cm × 10 cm) and two enclosed arms (50 cm × 10 cm, surrounded by a 40 cm-high black wall) elevated 50 cm above the floor. Rats were placed in the center area of the maze for each individual trial lasting 5 min. The number of entries and the time spent in the open arms were recorded and measured by an automatic analyzing system (Top Scan, Clever Sys Inc., United States) and used as indices of anxiety (Kim et al., 2016). The area was cleansed with 75% ethanol between tests.

### Fear Conditioning Test

The experiments were performed in the shock chamber (Context A: a rectangular box with stainless steel rod floors and aluminum and acrylic walls) and a neutral test context (Context B: a rectangular box with white acrylic floor and acrylic frame roof) as described previously (Nie et al., 2014; Liu et al., 2016). For shock application, rats were placed into the shock chamber (Context A) for 16 s and then exposed to a tone (3 min, 80 dB, 9 kHz); then, rats received a foot shock (4 s, 1.0 mA) and remained in the shock chamber for 60 s, after which they were returned to their home cages. The contextual fear conditioning test was performed 4 h after shock application. Rats were placed in the same chamber where they were trained (Context A) but without a tone or foot shock application for 3 min, and then they were immediately returned to their home cages. The auditory-cued fear test was performed 24 h later; rats were placed in the chamber (Context B) for 3 min, and received a neutral tone (3 min, 80 dB, 9 kHz). Then, rats remained in the test chamber (Context B) for another 60 s. The freezing behavior was recorded and analyzed by using a computerized automatic analyzing system (Freezing Scan, Clever Sys Inc., Reston, VA, United States).

### RNA Isolation and Quantitative Real-Time PCR (qRT-PCR) Analysis

Rat brains were rapidly dissected on ice after sacrificed and the hippocampus were isolated and placed on dry ice immediately. Then, total RNA was isolated by using the RNAiso Plus kit according to the manufacturer’s protocol (Takara Bio, Inc., Otsu, Japan). The quality and quantity of RNA were analyzed by spectrophotometry using the Multiskan Sky Microplate Spectrophotometer (Thermo Fisher Scientific, Inc.). The optical density at 260/280 nm of RNA in all samples ranged from 1.8 to 2.0 and the concentration ranged from 400 to 1,000 ng/μl. For real-time PCR analysis, 1,000 ng of RNA from each sample was reverse transcribed (37°C for 15 min, 85°C for 5 s, and 4°C for 10 min) by using the Prime-Script RT Reagent Kit (Takara Bio, Inc., Otsu, Japan). The cDNA was quantified by using real-time PCR with SYBR Premix Ex Taq^TM^ II (RR820Q, Takara) according to the manufacturer’s protocol on a Bio-Rad IQ5 Real-Time PCR Detection System. The primers used for real-time PCR were designed and synthesized by Takara Biotechnology Co., Ltd. (Dalian, China) according to the target mRNA sequence (*GAPDH*: NM_017008.4; *Nrf2*: NM_031789.2; and *keap1*: NM_057152.2). The primer sequences were as follows: *GAPDH*, forward and reverse: 5′-CCAATGTGTCCGTCGTGGATCT-3′ and 5′-GTTGAAGTCGCAGGAGACAACC-3′, respectively; *Nrf2*: forward and reverse, 5′-TTGGCAGAGACATTCCCA TTTGTA-3′ and 5′-GAGCTATCGAGTGACTGAGCCTGA-3′, respectively; and *keap1*: forward and reverse, 5′-CATCGGCATCGCCAACTTC-3′ and 5′-GCTGGCAGT GTGACAGGTTGA-3′, respectively. Each reaction consisted of 2 μl of cDNA product from the diluted reverse transcription reaction (5×), 0.5 μM of primers (forward and reverse), and 12.0 μl of SYBR Green real-time PCR master mix. The reactions were incubated in a 96-well plate and the two-step qRT-PCR program used was as follows: 1 cycle of 95°C for 30 s, followed by 40 cycles of 95°C for 5 s, 60°C for 30 s, and 1 cycle of 95°C for 15 s, and then maintained at 4°C. Subsequently, the relative changes in gene expression of Nrf2 and keap1 were normalized to the level of GAPDH mRNA of each sample (Jo et al., 2014; Chen et al., 2019) and analyzed by 2^-ΔΔCq^ method and shown relative to expression in control samples (Livak and Schmittgen, 2001).

### Lentivirus

A recombinant lentivirus coding for green fluorescent protein (GFP) carrying Nrf2-shRNA, keap1-shRNA or non-silencing RNA were purchased from Shanghai Genechem Co., Ltd. (Shanghai, China). Based on rat Nrf2 and keap1 mRNA sequences (accession number: NM_031789; NM_057152), three shRNA targeting different regions of Nrf2 mRNA (shNrf2-a, shNrf2-b, shNrf2-c), keap1 mRNA (shkeap1-a, shkeap1-b, shkeap1-c) and a scrambled non-silencing control shRNA (scramble) were generated. The targeting sequences were as follows: shNrf2-a, 5′-CTGGATGAAGAGACCGGAGAA-3’; shNrf2-b, 5′-GAGAAAGAATTGCCTGTAATT-3′; shNrf2-c, 5′-CCAAAGAGCAGTTCAATGATT-3′; shkeap1-a, 5′-GGA CAAACCGCCTTAATTCTT-3′; shkeap1-b, 5′-CAGCAGAACT GUACCTGTTTT-3′; shkeap1-1c, 5′-GGGCGTGGCTGTCCT CAATTT-3′; and scramble, 5′-TTCTCCGAACGTGTCACGT-3′.

### Primary Culture of Astrocytes and Transfection

As described previously (Zhou et al., 2017), astrocytes were harvested from the brains of newborn rats. Briefly, the hippocampus of newborn rats was isolated and single-cell suspensions were obtained. Then, the cells were resuspended in Dulbecco’s modified Eagle’s medium (DMEM) with 10% fetal bovine serum (FBS) and plated in 75-cm^2^ flasks coated with poly-L-lysine and incubated at 37°C with 5% CO_2_ for 10 days. After shaking at a speed of 240 rpm, astrocyte-enriched cultures were obtained. To screen and validate the efficiency of the produced lentivirus in targeting the Nrf2 and keap1 mRNAs, astrocytes were infected with the lentiviral particles according to the previous study (Li et al., 2012). Three days after transfection, the transfected astrocytes were harvested for real-time PCR and immunohistochemistry analysis.

To verify the identity of the astrocytes and the lentivirus infection, astrocytes were fixed in 4% paraformaldehyde at 4°C for 0.5 h. After washes in phosphate-buffered solution (PBS), the astrocytes were incubated with mouse anti-glial fibrillary acidic protein (GFAP, ab7260, 1:1,000, Abcam) diluted in buffer (1% w/v bovine serum albumin and 0.3% Triton in PBS), overnight at 4°C. The cells were washed and incubated with fluorescent secondary antibodies (Alexa Fluor 594 donkey anti-rabbit IgG, R37119, 1:1,000, Invitrogen) for 2 h and then incubated with DAPI for 20 min to stain the cellular nuclei. The preparations were analyzed under a laser-scanning confocal microscope (FV-1000, Olympus, Tokyo, Japan) and the silencing efficiency of shNrf2 and shkeap1 was assessed by real-time PCR. Ultimately, the shNrf2-a and shkeap1-c constructs were selected for the following *in vivo* experiments.

### Stereotaxic Surgery and Microinjections

As described previously (Uzakov et al., 2015), the concentrated titer-matched lentiviral suspension (5 μl, 2.5 μl for each side) was injected into the dentate gyrus (DG) (AP -3.0 mm; L ±1.8 mm; H 3.6 mm from dura) by an automatic nanoinjector at a rate of 0.25 μl/min. Then the syringe needle was left in position for 5 min after delivery to prevent reflux.

### Immunohistochemistry and Bromodeoxyuridine (BrdU) Detection

As described previously (Peng et al., 2018), rats were injected with 100 mg/kg BrdU (B5002, Sigma-Aldrich) for three consecutive days intraperitoneally. Twenty-four hours after the last BrdU injection, rats were anesthetized (chloral hydrate solution, i.p. 40 mg/kg) and then perfused with 4% paraformaldehyde in PBS. Brains were removed and transferred to 30% sucrose in PBS for 1 week to dehydrate and then sectioned (16-μm brain coronal sections) with a cryostat and mounted on gelatinized slides.

To assess cell proliferation, the brain sections were incubated in hydrochloric acid (2 N) at 37°C for 30 min and washed in 0.1 M sodium borate (pH 8.5) and PBS. Then, the sections were incubated with the primary antibody: anti-NeuN (ab177487, 1:500, Abcam) and anti-BrdU (B8434, 1:500, Sigma-Aldrich) at 4°C overnight. Next, they were incubated with secondary antibodies: Alexa Fluor 594 donkey anti-mouse (R37115, 1:1,000, Invitrogen) and Alexa Fluor 488 donkey anti-rabbit IgG (A-21206, 1:1,000, Invitrogen) or Alexa Fluor 405 goat anti-rabbit IgG (A-31556, 1:1,000, Invitrogen). The sections were observed under a fluorescence microscope and the BrdU-labeled cells were quantified.

For the immunofluorescence detection of DCX and cell-specific analysis of Nrf2, brain sections were incubated with primary antibody DCX (D9943, 1:1000, Sigma-Aldrich) and NeuN (ab177487, 1:500, Abcam), Nrf2 (AB413, 1:100, Sigma-Aldrich) and NeuN (MAB377B, 1:200, Sigma-Aldrich) or Nrf2 and GFAP (ab10062, 1:500, Abcam) overnight at 4°C after blocking with 5% (w/v) bovine serum albumin for 1 h. Subsequently, sections were incubated with Alexa Fluor 405 goat anti-rabbit IgG (A-31556, 1:1,000, Invitrogen) and Alexa Fluor 488 donkey anti-rabbit IgG (A-21206, 1:1,000, Invitrogen) or Alexa Fluor 594 donkey anti-rabbit IgG (R37115, 1:1,000, Invitrogen) for 2 h with or without DAPI at room temperature. The images were captured by the Olympus FV1200 confocal laser-scanning microscope (Olympus, Japan) and processed for further quantification. The percentage of double labeling of NeuN/Nrf2 and GFAP/Nrf2 was quantified by using Image-pro Plus 6.0 analysis software.

According to previous unbiased stereology protocol (Hill et al., 2018), every sixth section throughout the entire rostral caudal extent of the hippocampus was used to determine the number of BrdU-labeled cells or DCX^+^ in the DG. The number of BrdU+ or DCX+ cells was counted under a fluorescence microscope (Olympus, Japan) in the area of the subgranular zone (SGZ). The total number of positive cells in the SGZ of the hippocampal DG was estimated by multiplying the number of cells counted in every sixth section by six. For each marker, four animals were analyzed. All counts were performed by an experimenter blinded to the purpose of the study.

### Western Blot Analysis

Rat brains were rapidly dissected on ice after sacrificed and the hippocampus were isolated and washed with ice PBS. Then tissues were cut into pieces and weighed and lysed in a buffer composed of 62.5 mM Tris-HCl, 2% w/v sodium dodecyl sulfate, 10% glycerol, 50 mM dithiothreitol, and 0.1% w/v bromophenol blue. The protein concentrations of the supernatant were determined by the BCA Protein Assay Kit (Invitrogen). Then, samples were separated by 10% polyacrylamide gel (40 μg of total protein per lane) and transferred onto polyvinylidene difluoride membranes. The membranes were blocked with 5% non-fat dried milk and incubated with anti-Nrf2 (ab137550, 1:1000, Abcam), HO-1 (ab13248, 1:2000, Abcam), DCX (D9943, 1:1000, Sigma-Aldrich), AMPKα (2603, 1:1000, Cell Signaling), p-AMPKα (2535, 1: 1000, cell signaling), BDNF (ab205067, 1:1000, Abcam), keap1 (ab139729, 1:1000, Abcam), and β-actin antibodies (ab8227, 1:5000, Abcam) overnight at 4°C. The membranes were then washed and incubated with secondary antibodies for 1 h at room temperature. Immunoreactive bands were detected using the Super Signal West Pico Chemiluminescent Substrate (34077; Thermo Fisher Scientific, Inc.) and visualized on X-ray films. Quantifications were performed by using densitometric analysis implemented in the Bio-Rad QuantityOne1-D Analysis Software.

### Statistical Analyses

Data are presented as mean ± standard deviation and statistical analyses were performed by using SPSS 19.0 software (SPSS Inc., Chicago, IL, United States). Experimental data were subjected to Levene’s test and the Kolmogorov–Smirnov test for equality of variances and normal distribution, and then subjected to two- or one-way analysis of variance (ANOVA) with Tukey’s *post hoc* test was performed to compare means of different groups and *P* < 0.05 was defined as the threshold for statistically significance.

## Results

### EA Pretreatment Ameliorates Anxiety-Like and Fear Learning Behaviors in ESPS-Treated Rats

**FIGURE 1:**
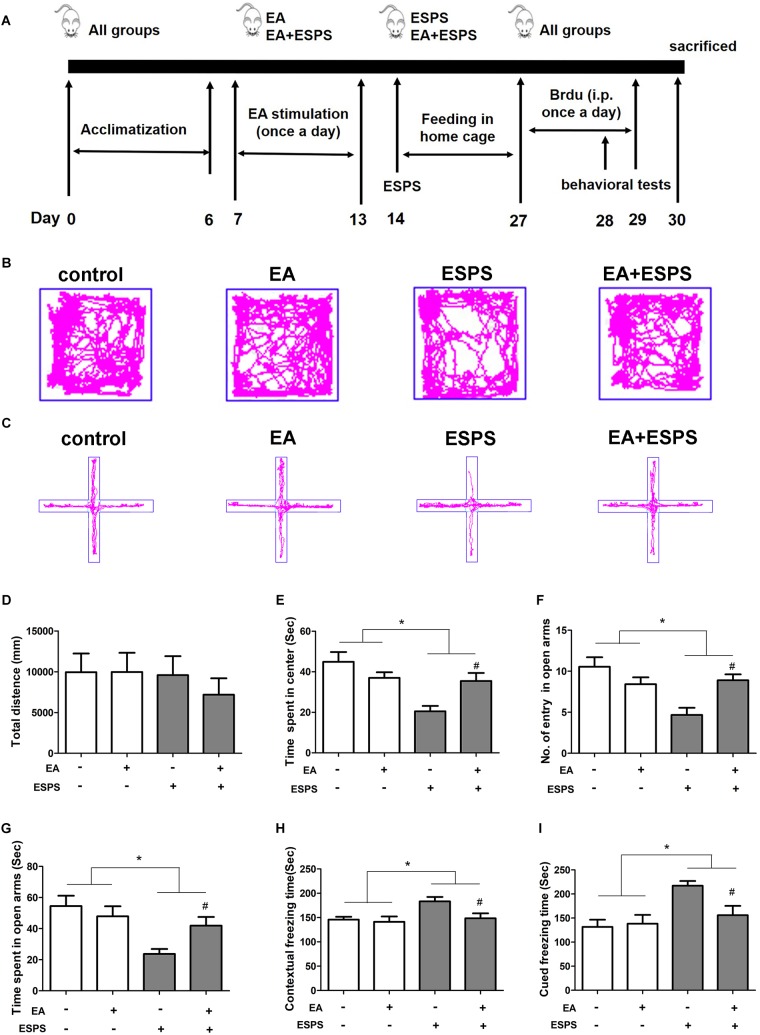
Electroacupuncture pretreatment ameliorates anxiety-like and fear learning behavior in ESPS-treated rats. **(A)** Timeline of the EA pretreatment, ESPS exposure, and behavioral testing in Experiment I. All animals were subjected to 1 week of adaptation, and then EA (EA at 1 mA in intensity and 2/15 Hz in frequency or EA without current) was administered once a day from days 7 through 13. ESPS treatment was performed on day 14. BrdU was administered once a day from days 27 through 29 by intraperitoneal injection and behavioral alterations were measured from days 28 through 30 before rats were sacrificed. **(B)** Real-time movement traces in the open field. **(C)** Elevated plus maze movement traces. **(D)** Quantification of the total distance traveled in the open field box. **(E)** The time spent in center of the open filed box. **(F)** Numbers of entries in the open arms of the elevated plus maze test. **(G)** The time spent in the open arms of the elevated plus maze test. **(H)** Freezing time of the contextual fear response. **(I)** Freezing time of the cued fear response. ^∗^*P* < 0.05, ^#^*P* < 0.05 vs. ESPS.

First, we determined the effect of EA pretreatment on PTSD (Figure 1A). Two-way ANOVA revealed that ESPS and EA treatment did not induce any motor impairment in rats because there were no differences in the total distance traveled in the OFT in both stress (control vs. ESPS, *F* = 0.486*, P* = 0.489) and EA treatment (control EA vs. EA, *F* = 0.275*, P* = 0.603) factors (Figure 1B,D). There were significant differences in the time spent in the center in the OFT for the stress factor (*F* = 13.606*, P* < 0.01, Figure 1E), as well as in the number of entries in the open arms (*F* = 8.532*, P* < 0.01) and the time spent in the open arms (*F* = 10.653, *P* < 0.01) in the EPMT (Figure 1C,F,G). However, there were no significant differences for the EA treatment factor neither in the time spent in the center in the OFT (*F* = 1.009*, P* = 0.321) nor as in the number of entries in the open arms (*F* = 1.298*, P* = 0.261) and the time spent in the open arms (*F* = 1.058, *P* = 0.310) in the EPMT. In addition, there were significant differences for the stress factor in the freezing times both during the contextual fear (*F* = 5.816*, P* < 0.05, Figure 1H) and the cued fear conditioning (*F* = 10.686*, P* < 0.05, Figure 1I) tests. Furthermore, there were also significant differences for the EA treatment factor in the freezing times during the contextual fear test (*F* = 4.474*, P* < 0.05). *Post hoc* comparisons further showed that ESPS markedly reduced the time spent in the center in the OFT as well as the number of entries into the open arms and the time spent in the open arms in the EPMT (ESPS vs. control, *P* < 0.05). Further, EA pretreatment increased values of these parameters (EA + ESPS vs. ESPS, *P* < 0.05). Additionally, rats in the ESPS group showed a significant increase of freezing time in both contextual fear and cued fear conditioning tests when compared to the control group (*P* < 0.05). EA pretreatment significantly decreased freezing times and enhanced fear learning in ESPS-treated rats (EA + ESPS vs. ESPS, *P* < 0.05, Figure 1H,I). These results suggest that EA pretreatment could ameliorate anxiety-like behaviors and fear learning in ESPS-treated rats.

### EA Pretreatment Increased Neurogenesis and BDNF Expression in the Hippocampus of ESPS-Treated Rats

**FIGURE 2:**
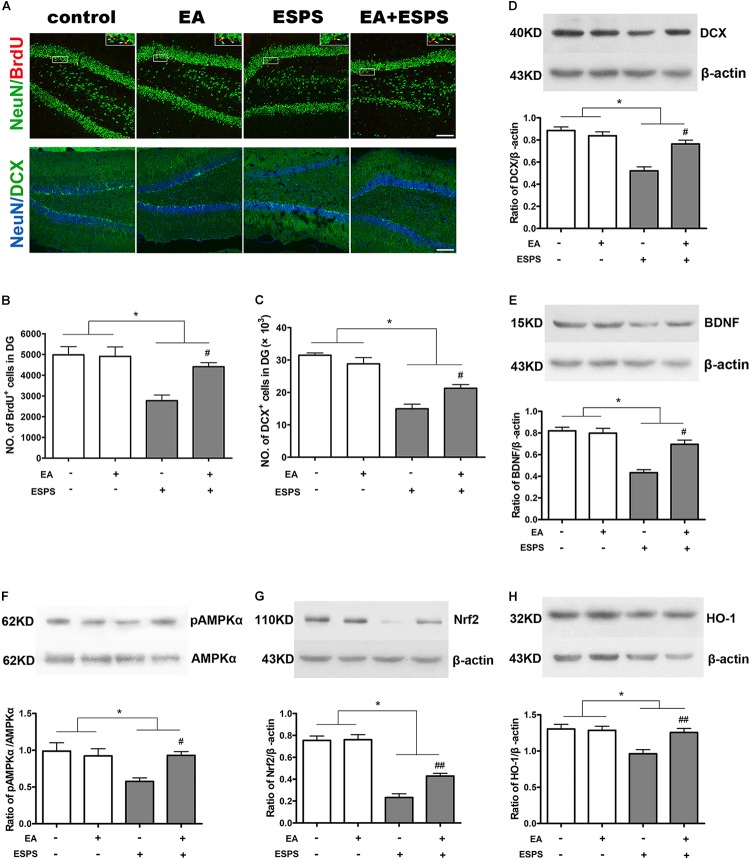
Electroacupuncture pretreatment improved hippocampal neurogenesis and the expression of BDNF, DCX, and Nrf2 and their related genes in ESPS-treated rats. **(A)** Microphotographs and **(B,C)** histograms of BrdU-positive proliferating cells and of the DCX-positive immature neurons in the dentate gyrus. **(D–H)** Representative immunoblots and densitometry analysis of **(D)** DCX, **(E)** BDNF, **(F)** p-AMPK, **(G)** Nrf2, and **(H)** HO-1 expression in the total hippocampus of control, EA, ESPS, and EA + ESPS groups. ^∗^*P* < 0.05, ^#^*P* < 0.05 vs. ESPS, ^##^*P* < 0.01 vs. ESPS. Bar: 100 μm.

As shown in Figure 2, there were significant differences for the stress factor in the number of BrdU-positive (BrdU^+^) (*F* = 15.455, *P* < 0.01, Figure 2A,B) and DCX-positive (DCX^+^) (*F* = 78.030, *P* < 0.01, Figure 2C) cells in the DG of the hippocampus. There were also significant differences for the EA treatment factor in the number of BrdU^+^ cells (*F* = 5.181, *P* < 0.05, Figure 2B) but not DCX^+^ cells (*F* = 1.256, *P* = 0.274, Figure 2C) in the DG. Meanwhile, there were significant differences of BDNF and DCX expression (Figure 2D,E) for both the stress (*F*_BDNF_ = 44.189*, P* < 0.01; *F*_DCX_ = 39.830*, P* < 0.01) and EA treatment (*F*_BDNF_ = 10.566*, P* < 0.01; *F*_DCX_ = 7.902*, P* < 0.05) factors. *Post hoc* comparisons further showed that ESPS decreased the number of BrdU^+^ and DCX^+^ cells in the hippocampus (ESPS vs. control, *P* < 0.01), and EA pretreatment prevented this damage induced by ESPS (EA + ESPS vs. ESPS, *P* < 0.05). In addition, ESPS stimulation markedly decreased the expression of DCX and BDNF (ESPS vs. control, *P* < 0.01), while EA pretreatment effectively reversed these changes (EA + ESPS vs. ESPS, *P* < 0.05). These results suggest that EA pretreatment is effective in preventing impairments of the hippocampal neurogenesis in ESPS-treated rats.

### EA Pretreatment Influences the Expression of Nrf2 and HO-1 and the Activity of AMPK in the Hippocampus of ESPS-Treated Rats

**FIGURE 3:**
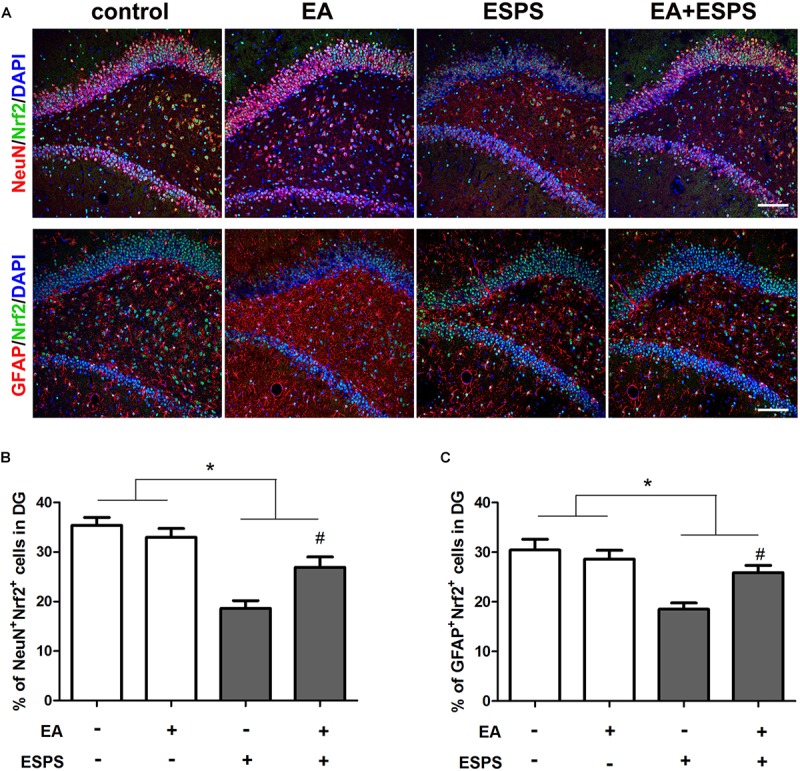
Electroacupuncture pretreatment improved hippocampal Nrf2 expression in both neurons and astrocytes in DG of ESPS-treated rats. **(A)** Microphotographs and **(B,C)** histograms of the percentage of NeuN/Nrf2 and GFAP/Nrf2 double labeling cells in the DG. ^∗^*P* < 0.05, ^#^*P* < 0.05 vs. ESPS. Bar: 100 μmm.

There were significant differences for the stress factor in the expression of Nrf2 (*F* = 13.443*, P* < 0.01, Figure 2G) and HO-1 (*F* = 10.367*, P* < 0.01, Figure 2H) and the activity of AMPK (*F* = 5.883*, P* < 0.05, Figure 2F). There were also significant differences for the EA treatment factor in the expression of Nrf2 (*F* = 7.487*, P* < 0.05) and HO-1 (*F* = 5.766*, P* < 0.05). However, there was no significant difference for the EA treatment factor in the activity of AMPK (*F* = 3.054*, P =* 0.094). Meanwhile, there were significant differences for the stress factor in the percentage of the NeuN^+^Nrf2^+^ (*F* = 41.861*, P* < 0.05, Figure 3A,B) and GFAP^+^Nrf2^+^ (*F* = 18.449*, P* < 0.05, Figure 3A,C) cells in DG. *Post hoc* comparisons further showed that ESPS decreased the activity of AMPK and the expression of Nrf2 and HO-1 (ESPS vs. control, *P* < 0.05), as well as the double labeling of NeuN and Nrf2 or GFAP and Nrf2, which were prevented by EA pretreatment (EA + ESPS vs. ESPS, *P* < 0.05). These results indicate that the neuroprotective effect of EA pretreatment in ESPS-treated rats could be mediated by the activation of AMPK/Nrf2 antioxidant pathway.

### Nrf2 Knockdown in the Adult Hippocampus Blocks the Protective Effects of EA Pretreatment on ESPS-Treated Rats

**FIGURE 4:**
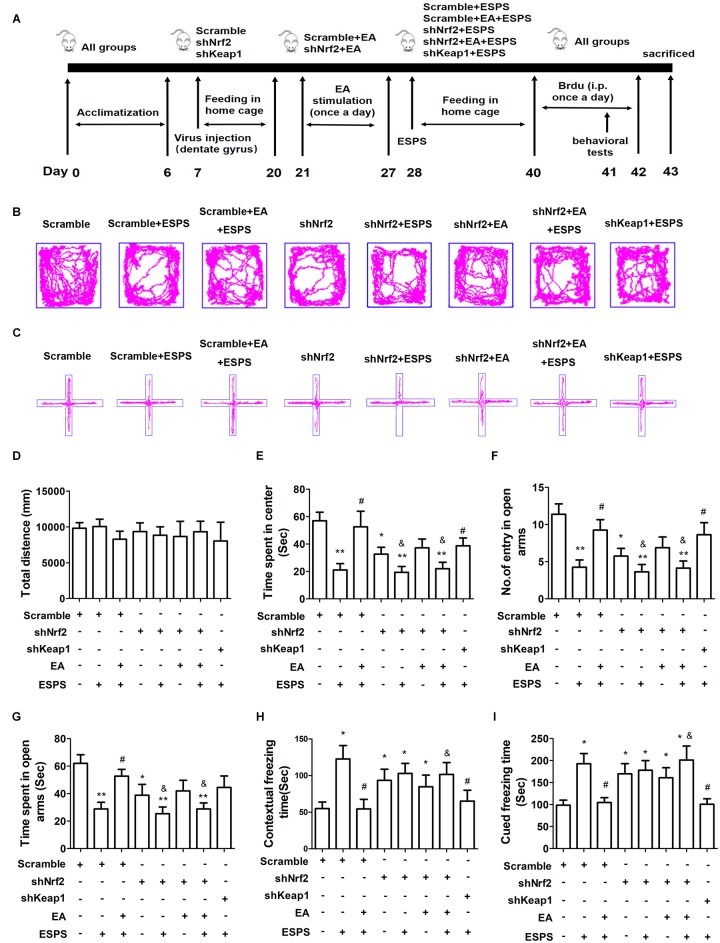
Nrf2 knockdown in the adult hippocampus inhibits the protective effects of electroacupuncture pretreatment on behavior of ESPS-treated rats. **(A)** Timeline of the stereotactic injection, EA pretreatment, ESPS administration and behavioral testing in Experiment II. Lentivirus with shRNA (Scramble or shNrf2 or shkeap1) were stereotaxically injected into the bilateral hippocampal dentate gyrus on day 7, EA was administered once a day from day 21 to 27. ESPS treatment was performed at day 28. BrdU was administered once a day from day 40 to 42 and behavioral alterations were measured from day 41 to 43 before rats were sacrificed. **(B)** Real-time movement traces in the open field. **(C)** Real-time movement traces in the Elevated plus maze. **(D)** Quantification of the total distance traveled in the open field test. **(E)** The time spent in center of the open filed box. **(F)** Numbers of entries in the open arms of the elevated plus maze test. **(G)** The time spent in the open arms of the elevated plus maze test. **(H)** Freezing times in the contextual fear. **(I)** Freezing times in the cued fear conditioning tests. ^∗^*P* < 0.05 vs. Scramble, ^∗∗^*P* < 0.01 vs. Scramble, ^#^*P* < 0.05 vs. Scramble + ESPS, ^&^*P* < 0.05 vs. Scramble + EA + ESPS.

In order to establish the contribution of Nrf2 on the effects of EA pretreatment (Figure 4A), we knocked down Nrf2 or Keap1 in the DG by bilateral injections of LV-GFP shRNA (shNrf2 or scramble) (Supplementary Figure S1). No significant differences in the total distance traveled in the open-field arena were observed (*F*_7,56_ = 0.210, *P* = 0.982, Figure 4B,D). However, there were significant differences in the time spent in the central area of the OFT (*F*_7,56_ = 4.937, *P* < 0.01, Figure 4E), the entry numbers (*F*_7,56_ = 4.938*, P* < 0.01, Figure 4C,F) and the time spent (*F*_7,56_ = 4.041, *P* < 0.01, Figure 4G) in the open arms of the EPMT, as well as the freezing time in the fear conditioning test (contextual freezing time: *F*_7,56_ = 2.831, *P* < 0.05, Figure 4H; cued freezing time *F*_7,56_ = 4.195, *P* < 0.01, Figure 4I). EA pretreatment successfully reversed all the changes associated to ESPS exposure (Scramble + EA + ESPS vs. Scramble + ESPS, *P* < 0.05). However, the protective effect of EA pretreatment was dampened by Nrf2 knockdown (shNrf2 + EA + ESPS vs. Scramble + EA + ESPS, *P* < 0.05). Moreover, downregulation of keap1 itself also ameliorated the deficits observed in behavior and in the hippocampal neurogenesis of ESPS-treated rats (shkeap1 + ESPS vs. Scramble + ESPS, *P* < 0.05). These results indicate that the keap1/Nrf2 antioxidant pathway may play a role in the anti-anxiety effects of EA pretreatment.

### The Neuroprotective Effect of EA Pretreatment Is Inhibited by Downregulation of Nrf2

**FIGURE 5:**
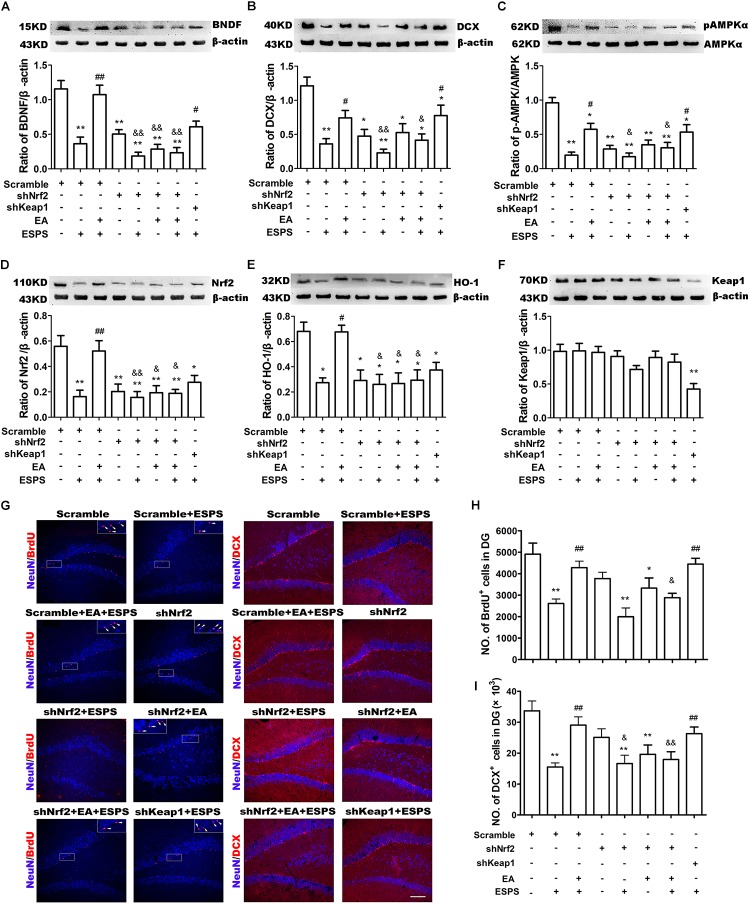
Knockdown of Nrf2 with shRNA lentivirus in the hippocampus dampened the neuroprotective effect of EA pretreatment on ESPS-treated rats. **(A–F)** Representative immunoblots and densitometry analysis of **(A)** BDNF, **(B)** DCX, **(C)** p-AMPK, **(D)** Nrf2, **(E)** HO-1, and **(F)** keap1 in the total hippocampus of Scramble, Scramble + ESPS, Scramble + EA + ESPS, shNrf2, shNrf2 + EA + ESPS, and shkeap1 + ESPS treated groups. **(G)** Microphotographs and **(H,I)** histograms of the BrdU-positive proliferation cells and of the DCX-positive immature neurons in the dentate gyrus. ^∗^*P* < 0.05 vs. Scramble; ^∗∗^*P* < 0.01 vs. Scramble; ^#^*P* < 0.05 vs. Scramble + ESPS; ^##^*P* < 0.01 vs. Scramble + ESPS; ^&^*P* < 0.05 vs. Scramble + EA + ESPS; ^&&^*P* < 0.01 vs. Scramble + EA + ESPS. Bar: 100 μm.

shNrf2 treatment effectively downregulated Nrf2 (*F*_7,24_ = 7.425, *P* < 0.01, Figure 5D) and HO-1 expression (*F*_7,24_ = 6.559, *P* < 0.01, Figure 5E), decreased AMPK (*F*_7,24_ = 12.33, *P* < 0.01, Figure 5C) activity, and dampened the effects of the EA pretreatment on the expression of BDNF (*F*_7,24_ = 16.85, *P* < 0.01, Figure 5A) and DCX (*F*_7,24_ = 7.484, *P* < 0.01, Figure 5B) in PTSD rats. In addition, shkeap1 significantly decreased keap1 protein level (Figure 5F) but increased BDNF and DCX expression in ESPS-treated rats, when compared to the Scramble + ESPS group (*P* < 0.05).

We also examined adult DG neurogenesis after lentiviral treatment. Significant differences in the number of BrdU^+^ (*F*_7,24_ = 8.057, *P* < 0.01, Figure 5G,H) and DCX^+^ (*F*_7,24_ = 6.339, *P* < 0.01, Figure 5G,I) cells were observed. EA pretreatment increased the number of BrdU^+^ and DCX^+^ cells (Scramble + EA + ESPS vs. Scramble + ESPS, *P* < 0.01). This effect was inhibited by Nrf2 downregulation (shNrf2 + EA + ESPS vs. Scramble + EA + ESPS, *P* < 0.05). Moreover, there were more BrdU^+^ and DCX^+^ cells in the shkeap1 + ESPS group than in the Scramble + ESPS group (*P* < 0.01).

## Discussion

In the present study, we provide first evidence that EA pretreatment can ameliorate the behavioral deficits and the impairments of hippocampus neurogenesis observed in ESPS-treated rats. EA pretreatment increased the expression of Nrf2, HO-1, and BDNF as well as the phosphorylation level of AMPK in the hippocampus of ESPS-treated rats. However, knockdown of Nrf2 in the hippocampus before the EA pretreatment dampened the therapeutic effects of the EA pretreatment, while keap1 knockdown in the hippocampus displayed similar neuro-protective effects to those observed for the EA pretreatment. We suggest that EA pretreatment may represent an effective preventive strategy for PTSD and its beneficial effects may involve the keap1/Nrf2 antioxidant pathway.

Acupuncture is a traditional Chinese medicine technique typically included in the field of complementary and alternative medicine (Langevin et al., 2011). It is widely used for managing chronic pain (Zhao et al., 2017; Hershman et al., 2018) and more recently has been suggested as a treatment for PTSD (Grant et al., 2018). The general sympatho-inhibitory effects of acupuncture depend on needle location and acupuncture type. Although research has not been as exhaustive as in manual acupuncture, EA has also been used in the treatment of several disorders as an improvement of traditional acupuncture. EA (particularly at low frequency) has been shown to produce more widespread effects in the brain than manual acupuncture, as assessed by functional magnetic resonance imaging (Napadow et al., 2005). Studies have found that EA influences the activity of the autonomic nervous system, as well as of prefrontal and limbic brain structures, including the amygdala, hippocampus, and the hypothalamus. EA has also been described to influence hypothalamic–pituitary–adrenal axis (HPA) function and plasma cortisol levels (Song et al., 2012; Mucuk et al., 2013; Le et al., 2016), which are involved in the pathophysiology of PTSD. In general, most of the preclinical and clinical studies on EA have focused on its role as a therapeutic agent. However, an ideal therapeutic scenario would involve prevention of symptoms even before its appearance. It is found that EA pretreatment (2/15 Hz) applied at the GV20 (“Bai hui”) conferred neuroprotection against cerebral ischemia (Wang et al., 2012; Zhao et al., 2015), and EA pretreatment with the same frequencies applied at Fengfu and Fengchi (GB20) provided neuroprotective effects during craniocerebral tumor resection (Lu et al., 2010). Meanwhile, 2/15 Hz EA pretreatment also reduced glutamate toxicity and exerted antiapoptotic effects on experimental stroke rats (Zhou et al., 2013; Zhu et al., 2013). More importantly, Wang et al. (2009) showed that EA pretreatment applied at the GV20 with 2/15 Hz conferred neuroprotection against cerebral ischemia by stimulating the production of 2-AG and AEA in the brain and activating CB1R. Consistent with the above implications, the present study indicates that EA pretreatment applied at the GV20 with 2/15 Hz for seven continuous days before rats are exposed to ESPS ameliorate PTSD-like behavior, including the increases in anxiety and the alterations in fear learning typically observed in these animals. Altogether, our data support the notion that EA pretreatment may be an effective therapy for the prevention of PTSD.

It is well known that hippocampal neurogenesis is involved in anxiety. Factors impairing hippocampal neurogenesis may induce disruption of mood and anxiety (Hill et al., 2015; Miller and Hen, 2015). In addition, hippocampal neurogenesis seems to be involved in anti-anxiety drug effects and promotion of hippocampal neurogenesis have been shown to hold the potential to alleviate anxiety and mood disorders (Jin et al., 2016; Mohammad et al., 2017). Recent anatomical and functional evidence indicates a dissociation of the dorsal and ventral regions of the hippocampus. It was found that the dorsal hippocampus is critical for learning and memory performance, while the ventral hippocampus is involved in anxiety and behavioral inhibition (Bannerman et al., 2014; Kempadoo et al., 2016; Floriou-Servou et al., 2018). In line with this, a growing body of evidences also supports a role for adult hippocampal neurogenesis in both the cognitive functions that are thought to be mediated by the dorsal hippocampus and emotional regulation that has been attributed to the ventral hippocampus (Wu and Hen, 2014; Zhang et al., 2018). Thus, neurogenesis in both the dorsal and ventral DG might be involved in the pathogenesis of PTSD. However, it should also be recognized that dorsal and ventral DG are not completely isolated from each other. Instead, they can interact via several routes (Fanselow and Dong, 2010). Meanwhile, it has been shown that hippocampal neurogenesis can be modulated indirectly by altering the *in vivo* hippocampal microenvironment (Monje et al., 2003; Seki, 2003). In our present study, although viral shRNA interference was delivered to dorsal DG, the decreased expression of Nrf2 or Keap1 was observed in the whole hippocampus (Supplementary Figure S1). The effects of viral shRNA interference that was delivered to other regions of the hippocampus were still needs further investigations.

Since Nrf2 is involved in the regulation of hippocampal neurogenesis and in the neuroprotective effects of EA (Wang X. R. et al., 2015; Robledinos-Anton et al., 2017), we further measured the activity of AMPK and expression of Nrf2 and HO-1 in the hippocampus. We also observed the involvement of Nrf2 or Keap1 in the neuroprotective effects of EA pretreatment by shRNA knockdown experiments. We showed that EA pretreatment promoted neurogenesis, as suggested by an increase in the number of BrdU^+^ cells and DCX^+^ immature neurons, and increased Nrf2/HO-1 and AMPK activity in the hippocampus of ESPS-treated rats. Downregulation of Nrf2 not only dampened the effects of EA pretreatment on PTSD-like behaviors but also reduced EA-induced increased neurogenesis in the hippocampus, indicating that the neuroprotective effects of EA pretreatment on PTSD rats may involve the Nrf2/HO-1 pathway. Previous studies have shown that the antioxidant effects of Nrf2 are exerted by disassociation from keap1, and treatment with a keap1 inhibitor exhibits protection in several diseases of the central nervous system (Quinti et al., 2017). In line with these results, we found that keap1 knockdown increased the expression of HO-1, improved hippocampal neurogenesis, and alleviated PTSD-like behaviors of ESPS-treated rats, replicating the therapeutic effects of EA. On the other hand, we found that Nrf2 knockdown alone induces effects that are similar to those of ESPS alone. Thus, it is a possible scenario that keap1/Nrf2 and its downstream antioxidative cascade elements play a role in the anti-PTSD effects of EA pretreatment. In addition, “Bai hui” is located on the skin incision line and the incision might interfere with the following EA treatment even after 2 weeks of recovery. Although the results in Experiment II indicated that ESPS induced a significant PTSD-like behavior in rats that received virus injection (Scramble vs. Scramble + ESPS), and this PTSD-like behavior was ameliorated by EA (Scramble + ESPS vs. Scramble + EA + ESPS), the interference of skin incision on the effects of EA pretreatment is still unrevealed. A comparison between EA + ESPS and Scramble + EA + ESPS is required in the future. In addition, Nrf2 is ubiquitously expressed in the central nervous system. Indeed, astrocytic-derived extrinsic support is known to play an important role in protecting neurons against oxidative stress (Shih et al., 2003). Previous studies further found that Nrf2-mediated glutathione biosynthesis and release from astrocytes protects neurons from oxidative stress, and Nrf2 overexpression specifically in astrocytes confers non-cell autonomous protection to surrounding neurons and leads to neuroprotection in *in vivo* models (Vargas et al., 2008; Chen et al., 2009). Recent work also indicated that developmental epigenetic Nrf2 repression weakens neuronal antioxidant defenses but is necessary to create an environment that supports neuronal development (Bell et al., 2015), and astrocytic Nrf2 signaling could be regulated by neuronal activity (Habas et al., 2013). Thus, based on the available literature, the regulation both on the expression of Nrf2 either in neurons or glial cells is meaningful. The present study found that Nrf2 was widely expressed in the nuclei of neurons and astrocytes, and ESPS induced a significant reduction in the double labeling of NeuN and Nrf2 or GFAP and Nrf2, which was ameliorated by EA pretreatment. The precise cellular mechanism still calls for further investigation.

In addition to reduced hippocampal neurogenesis, a growing body of evidence has been indicating that disruption of the brain derived neurotrophic factor (BDNF) may be also involved in the pathophysiology of PTSD (Cohen et al., 2018; Hou et al., 2018). Interestingly, recent studies have also reported that BDNF is involved in the biological effects of EA (Lin et al., 2017; Pak et al., 2018). The interplay between Nrf2 and BDNF has also been investigated. A previous study indicated that BDNF protein levels are decreased in the Nrf2 knockout mice (Martin-de-Saavedra et al., 2013). However, another study reported that Nrf2 activation was regulated by the TrkB-BDNF pathway (Bouvier et al., 2017) and the Nrf2 antioxidant axis was upregulated by BDNF overexpression in a rat model of traumatic brain injury (Chen et al., 2017; Ishii et al., 2018). In line with these observations, we found that EA pretreatment normalized BDNF expression in the hippocampus of ESPS rats, and this effect was blocked by Nrf2 shRNA knockdown. In addition, basal hippocampal expression of BDNF was also decreased in rats injected with Nrf2 shRNA, while down-regulation of keap1 up-regulated the expression of BDNF in the hippocampus of PTSD-like rats. Based on these data, we suggest that regulation of BDNF may also be involved in the anti-PTSD effects of EA pretreatment and that Nrf2 may be an upstream regulator of BDNF. However, we do not provide evidence clarifying the potential mechanism by which Nrf2 activation may regulate BDNF expression.

In summary, our results show that EA pretreatment has neuroprotective effects against ESPS-induced anxiety-like behaviors and hippocampal neurogenesis defects in rats. We also found that the neuroprotective effect of EA pretreatment was associated with an upregulation of the molecular mechanism associated with protection against oxidative damage and of BDNF expression. This effect of EA may involve the activation of the keap1/Nrf2/HO-1 pathway. Additionally, we found that Nrf2 is an upstream regulator of BDNF during EA-induced neuroprotection. Altogether, our findings provide new insights regarding the possibility of using EA in the prevention of PTSD and the mechanisms by which this protective effect may occur. However, the effects of different parameters of EA treatment on the activation of Nrf2 antioxidant pathway as well as the direct influence of Nrf2 knockout on PTSD-like behaviors remain unclear. Further studies are required to explore the detailed signaling cascades and cellular mechanisms involved in the regulation of keap1/Nrf2 after EA treatment.

## Ethics Statement

The experiment procedures were in accordance with the National Institutes of Health Guide for the Care and Use of Laboratory Animals and were approved by the Animal Use and Protection Committee of the Animal Center of Fourth Military Medical University.

## Author Contributions

Z-wP, H-nW, Q-rT, and Z-JZ were involved in conception and design of the study. C-hZ, FX, and S-sX conducted the final data analyses and drafted the manuscript. C-hZ, FX, LL, and S-sX acquired and analyzed the data. YW and MC performed Western blotting assay and analysis. Z-wP, H-fS, and Z-JZ reviewed the article. All authors have approved the final drafts of this manuscript for its publication.

## Conflict of Interest Statement

The authors declare that the research was conducted in the absence of any commercial or financial relationships that could be construed as a potential conflict of interest.

## Supplementary Material

FIGURE S1 Lentivirus screening and microinjections. The silencing efficiency of different lentiviruses carrying different shRNA sequences were tested in astrocyte cultures by real-time PCR analysis. **(A)** Representative microphotographs of GFAP staining (red), DAPI staining (blue), and lentivirus infection (green) in astrocytes. **(B,C)** Histograms showing the effect of different shRNA sequences on the levels of Nrf2 and keap1 mRNAs. **(D)** Microphotographs of the NeuN (blue) and lentivirus infection (green) signals in the dentate gyrus. **(E)** Illustration of the bilateral injection of the lentivirus in the dentate gyrus of rats. **(F,G)** Histograms showing the change of Nrf2 and keap1 mRNAs in the hippocampus of rats after lentivirus infection. **(H,I)** Representative immunoblots and densitometry analysis the change of Nrf2 and keap1 protein level in the hippocampus of rats after lentivirus infection. ^∗^*P* < 0.05 vs. Scramble; ^∗∗^*P* < 0.01 vs. Scramble. Bar: 100 m.

## References

[B1] Ahuja M. Ammal Kaidery N. Yang L. Calingasan N. Smirnova N. Gaisin A. (2016). Distinct Nrf2 signaling mechanisms of fumaric acid esters and their role in neuroprotection against 1-Methyl-4-Phenyl-1,2,3,6-tetrahydropyridine-induced experimental Parkinson’s-like disease. *J. Neurosci.* 36 6332–6351. 10.1523/jneurosci.0426-16.2016 27277809 PMC4899530

[B2] Bannerman D. M. Sprengel R. Sanderson D. J. Mchugh S. B. Rawlins J. N. Monyer H. (2014). Hippocampal synaptic plasticity, spatial memory and anxiety. *Nat. Rev. Neurosci.* 15 181–192. 24552786 10.1038/nrn3677

[B3] Bell K. F. Al-Mubarak B. Martel M. A. Mckay S. Wheelan N. Hasel P. (2015). Neuronal development is promoted by weakened intrinsic antioxidant defences due to epigenetic repression of Nrf2. *Nat. Commun.* 6:7066. 25967870 10.1038/ncomms8066 PMC4441249

[B4] Bouayed J. Rammal H. Soulimani R. (2009). Oxidative stress and anxiety: relationship and cellular pathways. *Oxid. Med. Cell Longev.* 2 63–67. 10.4161/oxim.2.2.7944 20357926 PMC2763246

[B5] Bouvier E. Brouillard F. Molet J. Claverie D. Cabungcal J. Cresto N. (2017). Nrf2-dependent persistent oxidative stress results in stress-induced vulnerability to depression. *Mol. Psychiatry* 22 1701–1713. 10.1038/mp.2016.144 27646262

[B6] Cai M. Tong L. Dong B. Hou W. Shi L. Dong H. (2017). Kelch-like ECH-associated protein 1-dependent nuclear factor-E2-related factor 2 activation in relation to antioxidation induced by sevoflurane preconditioning. *Anesthesiology* 126 507–521. 10.1097/aln.0000000000001485 28045693

[B7] Careaga M. B. Girardi C. E. Suchecki D. (2016). Understanding posttraumatic stress disorder through fear conditioning, extinction and reconsolidation. *Neurosci. Biobehav. Rev.* 71 48–57. 10.1016/j.neubiorev.2016.08.023 27590828

[B8] Chen P. C. Vargas M. R. Pani A. K. Smeyne R. J. Johnson D. A. Kan Y. W. (2009). Nrf2-mediated neuroprotection in the MPTP mouse model of Parkinson’s disease: critical role for the astrocyte. *Proc. Natl. Acad. Sci. U.S.A.* 106 2933–2938. 10.1073/pnas.0813361106 19196989 PMC2650368

[B9] Chen T. Wu Y. Wang Y. Zhu J. Chu H. Kong L. (2017). Brain-derived neurotrophic factor increases synaptic protein levels via the MAPK/Erk signaling pathway and Nrf2/Trx axis following the transplantation of neural stem cells in a rat model of traumatic brain injury. *Neurochem. Res.* 42 3073–3083. 10.1007/s11064-017-2340-7 28780733

[B10] Chen X. Wu Q. Chen Y. Zhang J. Li H. Yang Z. (2019). Diosmetin induces apoptosis and enhances the chemotherapeutic efficacy of paclitaxel in non-small cell lung cancer cells via Nrf2 inhibition. *Br. J. Pharmacol.* 176 2079–2094. 10.1111/bph.14652 30825187 PMC6534779

[B11] Chen Y. Lei Y. Mo L. Q. Li J. Wang M. H. Wei J. C. (2016). Electroacupuncture pretreatment with different waveforms prevents brain injury in rats subjected to cecal ligation and puncture via inhibiting microglial activation, and attenuating inflammation, oxidative stress and apoptosis. *Brain Res. Bull.* 127 248–259. 10.1016/j.brainresbull.2016.10.009 27771396

[B12] Chen Y. Zhou J. Li J. Yang S. B. Mo L. Q. Hu J. H. (2012). Electroacupuncture pretreatment prevents cognitive impairment induced by limb ischemia-reperfusion via inhibition of microglial activation and attenuation of oxidative stress in rats. *Brain Res.* 1432 36–45. 10.1016/j.brainres.2011.11.002 22129788

[B13] Cohen H. Kozlovsky N. Matar M. A. Zohar J. Kaplan Z. (2014). Distinctive hippocampal and amygdalar cytoarchitectural changes underlie specific patterns of behavioral disruption following stress exposure in an animal model of PTSD. *Eur. Neuropsychopharmacol.* 24 1925–1944. 10.1016/j.euroneuro.2014.09.009 25451698

[B14] Cohen H. Zohar J. Kaplan Z. Arnt J. (2018). Adjunctive treatment with brexpiprazole and escitalopram reduces behavioral stress responses and increase hypothalamic NPY immunoreactivity in a rat model of PTSD-like symptoms. *Eur. Neuropsychopharmacol.* 28 63–74. 10.1016/j.euroneuro.2017.11.017 29224968

[B15] Corcoran K. A. Maren S. (2001). Hippocampal inactivation disrupts contextual retrieval of fear memory after extinction. *J. Neurosci.* 21 1720–1726. 10.1523/jneurosci.21-05-01720.2001 11222661 PMC6762930

[B16] Fanselow M. S. Dong H. W. (2010). Are the dorsal and ventral hippocampus functionally distinct structures? *Neuron* 65 7–19. 10.1016/j.neuron.2009.11.031 20152109 PMC2822727

[B17] Feng S. Wang Q. Wang H. Peng Y. Wang L. Lu Y. (2010). Electroacupuncture pretreatment ameliorates hypergravity-induced impairment of learning and memory and apoptosis of hippocampal neurons in rats. *Neurosci. Lett.* 478 150–155. 10.1016/j.neulet.2010.05.006 20457216

[B18] Filipovic B. R. Djurovic B. Marinkovic S. Stijak L. Aksic M. Nikolic V. (2011). Volume changes of corpus striatum, thalamus, hippocampus and lateral ventricles in posttraumatic stress disorder (PTSD) patients suffering from headaches and without therapy. *Cent. Eur. Neurosurg.* 72 133–137. 10.1055/s-0030-1253349 20857373

[B19] Floriou-Servou A. Von Ziegler L. Stalder L. Sturman O. Privitera M. Rassi A. (2018). Distinct proteomic, transcriptomic, and epigenetic stress responses in dorsal and ventral hippocampus. *Biol. Psychiatry* 84 531–541. 10.1016/j.biopsych.2018.02.003 29605177

[B20] Gan L. Johnson J. A. (2014). Oxidative damage and the Nrf2-ARE pathway in neurodegenerative diseases. *Biochim. Biophys. Acta* 1842 1208–1218. 10.1016/j.bbadis.2013.12.011 24382478

[B21] Girardeau G. Inema I. Buzsaki G. (2017). Reactivations of emotional memory in the hippocampus-amygdala system during sleep. *Nat. Neurosci.* 20 1634–1642. 10.1038/nn.4637 28892057

[B22] Grant S. Colaiaco B. Motala A. Shanman R. Sorbero M. Hempel S. (2018). Acupuncture for the treatment of adults with posttraumatic stress disorder: a systematic review and meta-analysis. *J. Trauma Dissociation* 19 39–58. 10.1080/15299732.2017.1289493 28151093

[B23] Habas A. Hahn J. Wang X. Margeta M. (2013). Neuronal activity regulates astrocytic Nrf2 signaling. *Proc. Natl. Acad. Sci. U.S.A.* 110 18291–18296. 10.1073/pnas.1208764110 24145448 PMC3831500

[B24] Hershman D. L. Unger J. M. Greenlee H. Capodice J. L. Lew D. L. Darke A. K. (2018). Effect of acupuncture vs sham acupuncture or waitlist control on joint pain related to aromatase inhibitors among women with early-stage breast cancer: a randomized clinical trial. *JAMA* 320 167–176. 29998338 10.1001/jama.2018.8907 PMC6583520

[B25] Hill A. S. Sahay A. Hen R. (2015). Increasing adult hippocampal neurogenesis is sufficient to reduce anxiety and depression-like behaviors. *Neuropsychopharmacology* 40 2368–2378. 10.1038/npp.2015.85 25833129 PMC4538351

[B26] Hill J. D. Zuluaga-Ramirez V. Gajghate S. Winfield M. Persidsky Y. (2018). Activation of GPR55 increases neural stem cell proliferation and promotes early adult hippocampal neurogenesis. *Br. J. Pharmacol.* 175 3407–3421. 10.1111/bph.14387 29888782 PMC6057896

[B27] Hou L. Qi Y. Sun H. Wang G. Li Q. Wang Y. (2018). Applying ketamine to alleviate the PTSD-like effects by regulating the HCN1-related BDNF. *Prog. Neuropsychopharmacol. Biol. Psychiatry* 86 313–321. 10.1016/j.pnpbp.2018.03.019 29596995

[B28] Ishii T. Warabi E. Mann G. (2018). Circadian control of p75 neurotrophin receptor leads to alternate activation of Nrf2 and c-Rel to reset energy metabolism in astrocytes via brain-derived neurotrophic factor. *Free Radic. Biol. Med.* 119 34–44. 10.1016/j.freeradbiomed.2018.01.026 29374533

[B29] Jin J. Kim S. N. Liu X. Zhang H. Zhang C. Seo J. S. (2016). miR-17-92 cluster regulates adult hippocampal neurogenesis, anxiety, and depression. *Cell Rep.* 16 1653–1663. 10.1016/j.celrep.2016.06.101 27477270 PMC4981532

[B30] Jo C. Gundemir S. Pritchard S. Jin Y. N. Rahman I. Johnson G. V. (2014). Nrf2 reduces levels of phosphorylated tau protein by inducing autophagy adaptor protein NDP52. *Nat. Commun.* 5:3496. 24667209 10.1038/ncomms4496 PMC3990284

[B31] Kempadoo K. A. Mosharov E. V. Choi S. J. Sulzer D. Kandel E. R. (2016). Dopamine release from the locus coeruleus to the dorsal hippocampus promotes spatial learning and memory. *Proc. Natl. Acad. Sci. U.S.A.* 113 14835–14840. 10.1073/pnas.1616515114 27930324 PMC5187750

[B32] Khalifeh S. Oryan S. Digaleh H. Shaerzadeh F. Khodagholi F. Maghsoudi N. (2015). Involvement of Nrf2 in development of anxiety-like behavior by linking Bcl2 to oxidative phosphorylation: estimation in rat hippocampus, amygdala, and prefrontal cortex. *J. Mol. Neurosci.* 55 492–499. 10.1007/s12031-014-0370-z 25007950

[B33] Kheirbek M. A. Drew L. J. Burghardt N. S. Costantini D. O. Tannenholz L. Ahmari S. E. (2013). Differential control of learning and anxiety along the dorsoventral axis of the dentate gyrus. *Neuron* 77 955–968. 10.1016/j.neuron.2012.12.038 23473324 PMC3595120

[B34] Kim H. D. Hesterman J. Call T. Magazu S. Keeley E. Armenta K. (2016). SIRT1 mediates depression-like behaviors in the nucleus accumbens. *J. Neurosci.* 36 8441–8452. 10.1523/jneurosci.0212-16.2016 27511015 PMC4978803

[B35] Kubben N. Zhang W. Wang L. Voss T. C. Yang J. Qu J. (2016). Repression of the antioxidant NRF2 pathway in premature aging. *Cell* 165 1361–1374. 10.1016/j.cell.2016.05.017 27259148 PMC4893198

[B36] Langevin H. M. Wayne P. M. Macpherson H. Schnyer R. Milley R. M. Napadow V. (2011). Paradoxes in acupuncture research: strategies for moving forward. *Evid. Based. Complement. Alternat. Med.* 2011:180805. 20976074 10.1155/2011/180805 PMC2957136

[B37] Le J. J. Yi T. Qi L. Li J. Shao L. Dong J. C. (2016). Electroacupuncture regulate hypothalamic-pituitary-adrenal axis and enhance hippocampal serotonin system in a rat model of depression. *Neurosci. Lett.* 615 66–71. 10.1016/j.neulet.2016.01.004 26773866

[B38] Lee J. C. Kim I. H. Park J. H. Ahn J. H. Cho J. H. Cho G. S. (2015). Ischemic preconditioning protects hippocampal pyramidal neurons from transient ischemic injury via the attenuation of oxidative damage through upregulating heme oxygenase-1. *Free Radic. Biol. Med.* 79 78–90. 10.1016/j.freeradbiomed.2014.11.022 25483558

[B39] Li M. Husic N. Lin Y. Snider B. J. (2012). Production of lentiviral vectors for transducing cells from the central nervous system. *J. Vis. Exp.* 63:e4031. 10.3791/4031 PMC3466950 22664962

[B40] Lin J. Chen C. Yang H. Chen Y. Hung S. (2017). Electroacupuncture promotes recovery of motor function and reduces dopaminergic neuron degeneration in rodent models of Parkinson’s disease. *Int. J. Mol. Sci.* 18 E1846. 28837077 10.3390/ijms18091846 PMC5618495

[B41] Liu F. F. Yang L. D. Sun X. R. Zhang H. Pan W. Wang X. M. (2016). NOX2 mediated-parvalbumin interneuron loss might contribute to anxiety-like and enhanced fear learning behavior in a rat model of post-traumatic stress disorder. *Mol. Neurobiol.* 53 6680–6689. 10.1007/s12035-015-9571-x 26650043

[B42] Liu H. Petukhova M. V. Sampson N. A. Aguilar-Gaxiola S. Alonso J. Andrade L. H. (2017). Association of DSM-IV posttraumatic stress disorder with traumatic experience type and history in the world health organization world mental health surveys. *JAMA Psychiatry* 74 270–281. 28055082 10.1001/jamapsychiatry.2016.3783 PMC5441566

[B43] Livak K. J. Schmittgen T. D. (2001). Analysis of relative gene expression data using real-time quantitative PCR and the 2(-Delta Delta C(T)) Method. *Methods* 25 402–408. 10.1006/meth.2001.1262 11846609

[B44] Lu Z. H. Bai X. G. Xiong L. Z. Wang Y. H. Wang Y. Wang Q. (2010). Effect of electroacupuncture preconditioning on serum S100beta and NSE in patients undergoing craniocerebral tumor resection. *Chin. J. Integr. Med.* 16 229–233. 10.1007/s11655-010-0229-6 20694777

[B45] Martin-de-Saavedra M. D. Budni J. Cunha M. P. Gomez-Rangel V. Lorrio S. Del Barrio L. (2013). Nrf2 participates in depressive disorders through an anti-inflammatory mechanism. *Psychoneuroendocrinology* 38 2010–2022. 10.1016/j.psyneuen.2013.03.020 23623252

[B46] McLaughlin K. A. Koenen K. C. Friedman M. J. Ruscio A. M. Karam E. G. Shahly V. (2015). Subthreshold posttraumatic stress disorder in the world health organization world mental health surveys. *Biol. Psychiatry* 77 375–384. 24842116 10.1016/j.biopsych.2014.03.028 PMC4194258

[B47] Miller B. R. Hen R. (2015). The current state of the neurogenic theory of depression and anxiety. *Curr. Opin. Neurobiol.* 30 51–58. 10.1016/j.conb.2014.08.012 25240202 PMC4293252

[B48] Miller M. W. Sadeh N. (2014). Traumatic stress, oxidative stress and post-traumatic stress disorder: neurodegeneration and the accelerated-aging hypothesis. *Mol. Psychiatry* 19 1156–1162. 10.1038/mp.2014.111 25245500 PMC4211971

[B49] Missault S. Anckaerts C. Blockx I. Deleye S. Van Dam D. Barriche N. (2019). Neuroimaging of subacute brain inflammation and microstructural changes predicts long-term functional outcome after experimental traumatic brain injury. *J. Neurotrauma* 36 768–788. 10.1089/neu.2018.5704 30032713

[B50] Mitsuishi Y. Motohashi H. Yamamoto M. (2012). The Keap1-Nrf2 system in cancers: stress response and anabolic metabolism. *Front. Oncol.* 2:200. 10.3389/fonc.2012.00200 23272301 PMC3530133

[B51] Mohammad H. Marchisella F. Ortega-Martinez S. Hollos P. Eerola K. Komulainen E. (2017). JNK1 controls adult hippocampal neurogenesis and imposes cell-autonomous control of anxiety behaviour from the neurogenic niche. *Mol. Psychiatry* 23 362–374. 10.1038/mp.2016.203 27843149 PMC5794884

[B52] Monje M. L. Toda H. Palmer T. D. (2003). Inflammatory blockade restores adult hippocampal neurogenesis. *Science* 302 1760–1765. 10.1126/science.1088417 14615545

[B53] Moustafa A. A. (2013). Increased hippocampal volume and gene expression following cognitive behavioral therapy in PTSD. *Front. Hum. Neurosci.* 7:747. 10.3389/fnhum.2013.00747 24223547 PMC3819529

[B54] Mucuk S. Baser M. Ozkan T. (2013). Effects of noninvasive electroacupuncture on labor pain, adrenocorticotropic hormone, and cortisol. *Altern. Ther. Health Med.* 19 26–30. 23709457

[B55] Napadow V. Makris N. Liu J. Kettner N. W. Kwong K. K. Hui K. K. (2005). Effects of electroacupuncture versus manual acupuncture on the human brain as measured by fMRI. *Hum. Brain Mapp.* 24 193–205. 10.1002/hbm.20081 15499576 PMC6871725

[B56] Nie H. Peng Z. Lao N. Wang H. Chen Y. Fang Z. (2014). Rosmarinic acid ameliorates PTSD-like symptoms in a rat model and promotes cell proliferation in the hippocampus. *Prog. Neuropsychopharmacol. Biol. Psychiatry* 51 16–22. 10.1016/j.pnpbp.2014.01.002 24418162

[B57] Pak M. E. Jung D. H. Lee H. J. Shin M. J. Kim S. Y. Shin Y. B. (2018). Combined therapy involving electroacupuncture and treadmill exercise attenuates demyelination in the corpus callosum by stimulating oligodendrogenesis in a rat model of neonatal hypoxia-ischemia. *Exp. Neurol.* 300 222–231. 10.1016/j.expneurol.2017.11.014 29199131

[B58] Peng Z. Deng B. Jia J. Hou W. Hu S. Deng J. (2018). Liver X receptor beta in the hippocampus: a potential novel target for the treatment of major depressive disorder? *Neuropharmacology* 135 514–528. 10.1016/j.neuropharm.2018.04.014 29654801

[B59] Peng Z. Zhang R. Wang H. Chen Y. Xue F. Wang L. (2013). Ziprasidone ameliorates anxiety-like behaviors in a rat model of PTSD and up-regulates neurogenesis in the hippocampus and hippocampus-derived neural stem cells. *Behav. Brain Res.* 244 1–8. 10.1016/j.bbr.2013.01.032 23384713

[B60] Qiu Y. L. Cheng X. N. Bai F. Fang L. Y. Hu H. Z. Sun D. Q. (2018). Aucubin protects against lipopolysaccharide-induced acute pulmonary injury through regulating Nrf2 and AMPK pathways. *Biomed. Pharmacother.* 106 192–199. 10.1016/j.biopha.2018.05.070 29958143

[B61] Quinti L. Dayalan Naidu S. Trager U. Chen X. Kegel-Gleason K. Lleres D. (2017). KEAP1-modifying small molecule reveals muted NRF2 signaling responses in neural stem cells from Huntington’s disease patients. *Proc. Natl. Acad. Sci. U.S.A.* 114 E4676–E4685. 28533375 10.1073/pnas.1614943114 PMC5468652

[B62] Robledinos-Anton N. Rojo A. I. Ferreiro E. Nunez A. Krause K. H. Jaquet V. (2017). Transcription factor NRF2 controls the fate of neural stem cells in the subgranular zone of the hippocampus. *Redox Biol.* 13 393–401. 10.1016/j.redox.2017.06.010 28667908 PMC5493838

[B63] Seki T. (2003). Microenvironmental elements supporting adult hippocampal neurogenesis. *Anat. Sci. Int.* 78 69–78. 10.1046/j.0022-7722.2003.00043.x 12828419

[B64] Shih A. Y. Johnson D. A. Wong G. Kraft A. D. Jiang L. Erb H. (2003). Coordinate regulation of glutathione biosynthesis and release by Nrf2-expressing glia potently protects neurons from oxidative stress. *J. Neurosci.* 23 3394–3406. 10.1523/jneurosci.23-08-03394.2003 12716947 PMC6742304

[B65] Song J. G. Li H. H. Cao Y. F. Lv X. Zhang P. Li Y. S. (2012). Electroacupuncture improves survival in rats with lethal endotoxemia via the autonomic nervous system. *Anesthesiology* 116 406–414. 10.1097/aln.0b013e3182426ebd 22222470

[B66] Sripada R. K. Rauch S. A. Liberzon I. (2016). Psychological mechanisms of PTSD and Its Treatment. *Curr. Psychiatry Rep.* 18:99. 10.1007/s11920-016-0735-9 27671916

[B67] Sullivan G. M. Apergis J. Gorman J. M. Ledoux J. E. (2003). Rodent doxapram model of panic: behavioral effects and c-Fos immunoreactivity in the amygdala. *Biol. Psychiatry* 53 863–870. 10.1016/s0006-3223(02)01733-x 12742673

[B68] Uzakov S. S. Ivanov A. D. Salozhin S. V. Markevich V. A. Gulyaeva N. V. (2015). Lentiviral-mediated overexpression of nerve growth factor (NGF) prevents beta-amyloid [25-35]-induced long term potentiation (LTP) decline in the rat hippocampus. *Brain Res.* 1624 398–404. 10.1016/j.brainres.2015.07.051 26254730

[B69] Vargas M. R. Johnson D. A. Sirkis D. W. Messing A. Johnson J. A. (2008). Nrf2 activation in astrocytes protects against neurodegeneration in mouse models of familial amyotrophic lateral sclerosis. *J. Neurosci.* 28 13574–13581. 10.1523/jneurosci.4099-08.2008 19074031 PMC2866507

[B70] Wang F. Zhong H. Li X. Peng Y. Kinden R. Liang W. (2014). Electroacupuncture attenuates reference memory impairment associated with astrocytic NDRG2 suppression in APP/PS1 transgenic mice. *Mol. Neurobiol.* 50 305–313. 10.1007/s12035-013-8609-1 24390566

[B71] Wang H. N. Bai Y. H. Chen Y. C. Zhang R. G. Wang H. H. Zhang Y. H. (2015). Repetitive transcranial magnetic stimulation ameliorates anxiety-like behavior and impaired sensorimotor gating in a rat model of post-traumatic stress disorder. *PLoS One* 10:e0117189. 10.1371/journal.pone.0117189 25659132 PMC4320076

[B72] Wang X. R. Shi G. X. Yang J. W. Yan C. Q. Lin L. T. Du S. Q. (2015). Acupuncture ameliorates cognitive impairment and hippocampus neuronal loss in experimental vascular dementia through Nrf2-mediated antioxidant response. *Free Radic. Biol. Med.* 89 1077–1084. 10.1016/j.freeradbiomed.2015.10.426 26546103

[B73] Wang Q. Peng Y. Chen S. Gou X. Hu B. Du J. (2009). Pretreatment with electroacupuncture induces rapid tolerance to focal cerebral ischemia through regulation of endocannabinoid system. *Stroke* 40 2157–2164. 10.1161/strokeaha.108.541490 19372445

[B74] Wang Q. Wang F. Li X. Yang Q. Li X. Xu N. (2012). Electroacupuncture pretreatment attenuates cerebral ischemic injury through alpha7 nicotinic acetylcholine receptor-mediated inhibition of high-mobility group box 1 release in rats. *J. Neuroinflamm.* 9:24. 22277256 10.1186/1742-2094-9-24 PMC3297509

[B75] Wilson C. B. Mclaughlin L. D. Nair A. Ebenezer P. J. Dange R. Francis J. (2013). Inflammation and oxidative stress are elevated in the brain, blood, and adrenal glands during the progression of post-traumatic stress disorder in a predator exposure animal model. *PLoS One* 8:e76146. 10.1371/journal.pone.0076146 24130763 PMC3794007

[B76] Wu M. V. Hen R. (2014). Functional dissociation of adult-born neurons along the dorsoventral axis of the dentate gyrus. *Hippocampus* 24 751–761. 10.1002/hipo.22265 24550158 PMC4222246

[B77] Yang H. Yang J. Xi W. Hao S. Luo B. He X. (2016). Laterodorsal tegmentum interneuron subtypes oppositely regulate olfactory cue-induced innate fear. *Nat. Neurosci.* 19 283–289. 10.1038/nn.4208 26727549

[B78] Yu J. B. Shi J. Gong L. R. Dong S. A. Xu Y. Zhang Y. (2014). Role of Nrf2/ARE pathway in protective effect of electroacupuncture against endotoxic shock-induced acute lung injury in rabbits. *PLoS One* 9:e104924. 10.1371/journal.pone.0104924 25115759 PMC4130631

[B79] Yu J. B. Shi J. Zhang Y. Gong L. R. Dong S. A. Cao X. S. (2015). Electroacupuncture ameliorates acute renal injury in lipopolysaccharide-stimulated rabbits via induction of HO-1 through the PI3K/Akt/Nrf2 Pathways. *PLoS One* 10:e0141622. 10.1371/journal.pone.0141622 26524181 PMC4629879

[B80] Yuan T. F. Gu S. Shan C. Marchado S. Arias-Carrion O. (2015). Oxidative stress and adult neurogenesis. *Stem. Cell Rev.* 11 706–709. 10.1007/s12015-015-9603-y 26100529

[B81] Zhang T. Y. Keown C. L. Wen X. Li J. Vousden D. A. Anacker C. (2018). Environmental enrichment increases transcriptional and epigenetic differentiation between mouse dorsal and ventral dentate gyrus. *Nat. Commun.* 9 298. 29352183 10.1038/s41467-017-02748-x PMC5775256

[B82] Zhao L. Chen J. Li Y. Sun X. Chang X. Zheng H. (2017). The long-term effect of acupuncture for migraine prophylaxis: a randomized clinical trial. *JAMA Intern. Med.* 177 508–515. 28241154 10.1001/jamainternmed.2016.9378

[B83] Zhao Y. Deng B. Li Y. Zhou L. Yang L. Gou X. (2015). Electroacupuncture pretreatment attenuates cerebral ischemic injury via notch pathway-mediated up-regulation of hypoxia inducible factor-1alpha in rats. *Cell Mol. Neurobiol.* 35 1093–1103. 10.1007/s10571-015-0203-9 25976178 PMC4602051

[B84] Zhou C. H. Zhang Y. H. Xue F. Xue S. S. Chen Y. C. Gu T. (2017). Isoflurane exposure regulates the cell viability and BDNF expression of astrocytes via upregulation of TREK-1. *Mol. Med. Rep.* 16 7305–7314. 10.3892/mmr.2017.7547 28944872 PMC5865860

[B85] Zhou H. Zhang Z. Wei H. Wang F. Guo F. Gao Z. (2013). Activation of STAT3 is involved in neuroprotection by electroacupuncture pretreatment via cannabinoid CB1 receptors in rats. *Brain Res.* 1529 154–164. 10.1016/j.brainres.2013.07.006 23880371

[B86] Zhu X. Yin J. Li L. Ma L. Tan H. Deng J. (2013). Electroacupuncture preconditioning-induced neuroprotection may be mediated by glutamate transporter type 2. *Neurochem. Int.* 63 302–308. 10.1016/j.neuint.2013.06.017 23831620 PMC3758789

