# Supplementary figures and images for: Electroacupuncture Pretreatment Ameliorates PTSD-Like Behaviors in Rats by Enhancing Hippocampal Neurogenesis via the Keap1/Nrf2 Antioxidant Signaling Pathway
Source: Front Cell Neurosci. 2019 Jun 21;13:275. doi: 10.3389/fncel.2019.00275 (PMC6598452; doi:10.3389/fncel.2019.00275)

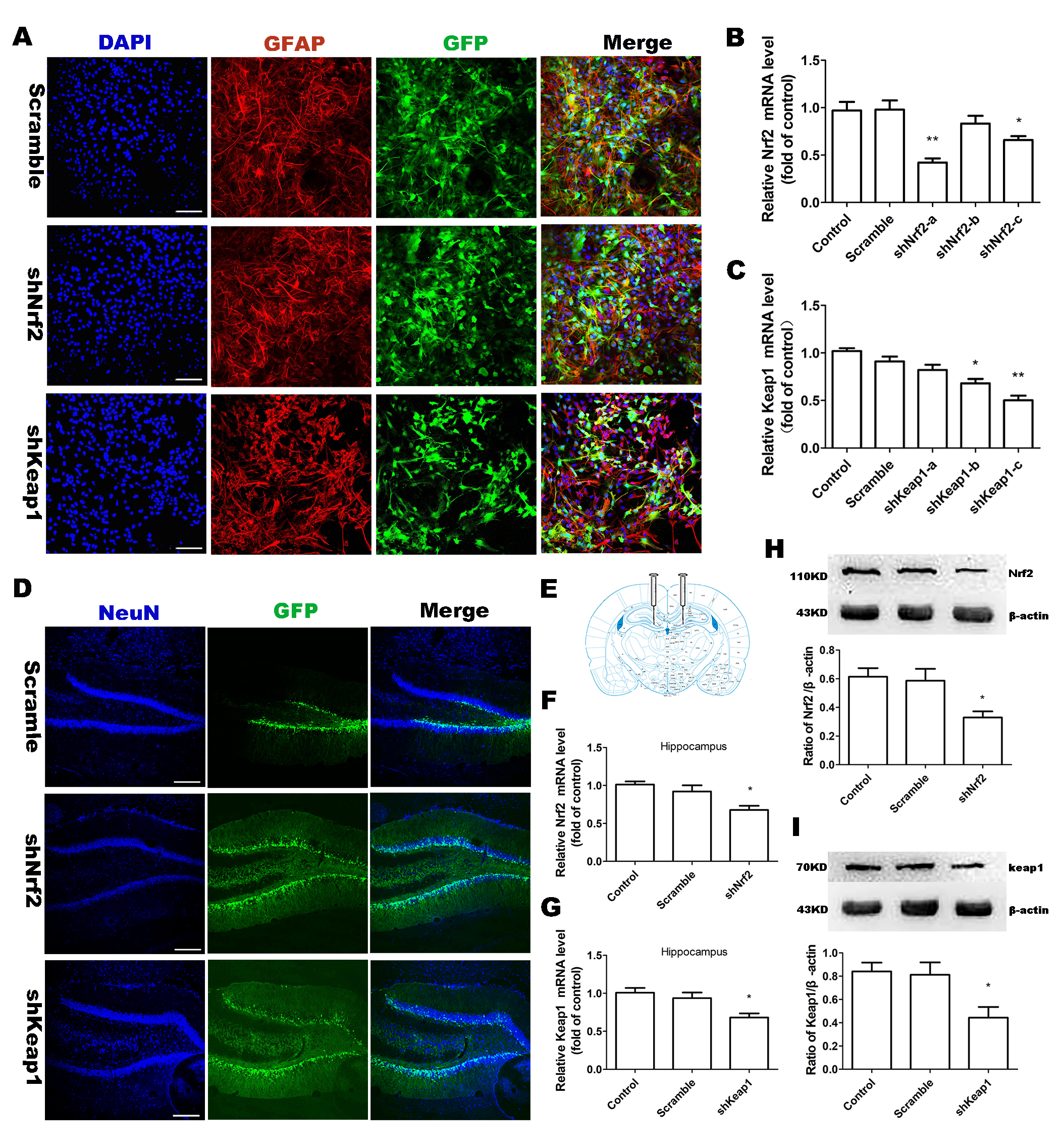

Supplement: FIGURE S1 — Lentivirus screening and microinjections. The silencing efficiency of different lentiviruses carrying different shRNA sequences were tested in astrocyte cultures by real-time PCR analysis. (A) Representative microphotographs of GFAP staining (red), DAPI staining (blue), and lentivirus infection (green) in astrocytes. (B,C) Histograms showing the effect of different shRNA sequences on the levels of Nrf2 and keap1 mRNAs. (D) Microphotographs of the NeuN (blue) and lentivirus infection (green) signals in the dentate gyrus. (E) Illustration of the bilateral injection of the lentivirus in the dentate gyrus of rats. (F,G) Histograms showing the change of Nrf2 and keap1 mRNAs in the hippocampus of rats after lentivirus infection. (H,I) Representative immunoblots and densitometry analysis the change of Nrf2 and keap1 protein level in the hippocampus of rats after lentivirus infection. ∗P < 0.05 vs. Scramble; ∗∗P < 0.01 vs. Scramble. Bar: 100 m. [file Image_1.JPEG]
